# Using a conceptual framework during learning attenuates the loss of expert-type knowledge structure

**DOI:** 10.1186/1472-6920-6-37

**Published:** 2006-07-18

**Authors:** Kerry Novak, Henry Mandin, Elizabeth Wilcox, Kevin McLaughlin

**Affiliations:** 1 University of Calgary, Calgary, Alberta, Canada

## Abstract

**Background:**

During evolution from novice to expert, knowledge structure develops into an abridged network organized around pathophysiological concepts. The objectives of this study were to examine the change in knowledge structure in medical students in one year and to investigate the association between the use of a conceptual framework (diagnostic scheme) and long-term knowledge structure.

**Methods:**

Medical students' knowledge structure of metabolic alkalosis was studied after instruction and one year later using concept-sorting. Knowledge structure was labeled 'expert-type' if students shared ≥ 2 concepts with experts and 'novice-type' if they shared < 2 concepts. Conditional logistic regression was used to study the association between short-term knowledge structure, the use of a diagnostic scheme and long-term knowledge structure.

**Results:**

Thirty-four medical students completed the concept-sorting task on both occasions. Twenty-four used a diagnostic scheme for metabolic alkalosis. Short-term knowledge structure was not a correlate of long-term knowledge structure, whereas use of a diagnostic scheme was associated with increased odds of expert-type long-term knowledge structure (odds ratio 12.6 [1.4, 116.0], p = 0.02). There was an interaction between short-term knowledge structure and the use of a diagnostic scheme. In the group who did not use a diagnostic scheme the number of students changing from expert-type to novice-type was greater than vice versa (p = 0.046). There was no significant change in the group that used the diagnostic scheme (p = 0.6).

**Conclusion:**

The use of a diagnostic scheme by students may attenuate the loss of expert-type knowledge structure.

## Background

In medical problem solving, information learned in the setting of a previous problem is [hopefully] transferred to a new problem [1]. Medical knowledge is thought to be stored in a network structure and connections between items made on the basis of both pathophysiological and clinical associations [2,3]. During the evolution from novice to expert a large amount of new information about diseases is incorporated into the knowledge network and the structure is thought to evolve into an abridged network within which knowledge items are encapsulated into higher order concepts [4]. The belief that formation of higher order concepts in knowledge structure is an integral step in the pathway to development of expertise has spawned research aimed at both evaluating and facilitating the development of these concepts. In the area of evaluation, for example, analysis of the interrelationship between concepts within a knowledge network (concept mapping analysis, CMA) has been proposed as a tool for evaluating both pre-existing knowledge and knowledge gained as a result of an educational intervention [5,6]. An important assumption [and criticism] of CMA is that the map generated represents the 'true' knowledge structure of the subject rather than merely a creation of the research tool; a hypothesis that is difficult to prove. The observation, however, that the map generated by CMA correlates with performance provides some support for this assumption upon which CMA is based [7,8].

In a previous study using concept-sorting as an analysis tool, it was found that medical students who shared more concepts with experts, i.e., with 'expert-type' [or 'deep'] knowledge structure, were more likely to be successful at problem solving than students with 'novice-type' [or 'surface'] knowledge structure [7]. This initial report was in a single clinical presentation, metabolic alkalosis, but has now also been observed in three other presentations in the clinical presentation of nephrology (metabolic acidosis, hyponatremia and hyperkalemia) [9]. In the initial report, it was also observed that students who used a conceptual framework, in the form of a diagnostic scheme, to organize learning were more likely to have expert-type knowledge structure [7]. In the subsequent study, it was observed that when preceptors used diagnostic schemes during the learning experiences, students were more likely to have expert-type knowledge structure [9]. These studies suggest that the development of expert-type knowledge structure may be facilitated in students by the explicit use of diagnostic schemes during learning and teaching.

**Figure 1:**
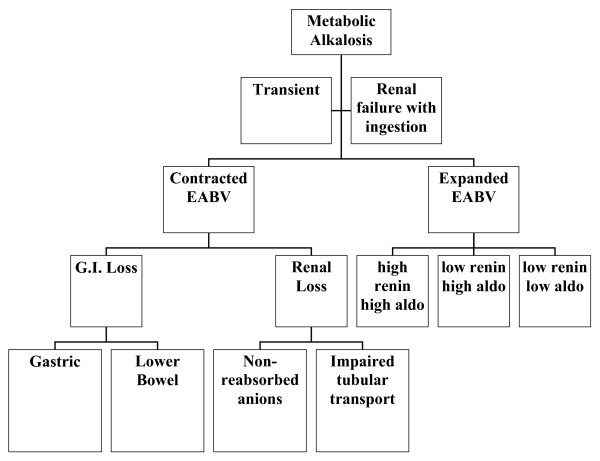
Conceptual framework (diagnostic scheme) for the clinical presentation of metabolic alkalosis.

A diagnostic scheme is a type of hierarchal conceptual framework where the relationship between the upper and lower levels can be categorized as 'subsumes' [10]. The diagnostic scheme for metabolic alkalosis is shown in Figure 1. At the University of Calgary the curriculum is divided into 125 ± 5 clinical presentations and for each presentation a diagnostic scheme is given to students to help organize their learning [11,12]. The concepts in the diagnostic schemes and the interrelationship between these concepts were based on the results of CMA of experts in each clinical presentation. While students are encouraged to use and/or modify the diagnostic schemes, the utilization by students during learning is variable. In a previous study, the frequency of diagnostic scheme use by students ranged from 57% to 90% depending upon the clinical presentation [7]. Seventy two percent of students used the diagnostic scheme for metabolic alkalosis.

It has previously been shown in education research in children that when students are provided with a conceptual framework, knowledge transfer is enhanced [13]. In medical education it has also been shown that teaching around pathophysiological concepts, as compared to clinical probability, increases the likelihood of diagnostic success one week after instruction despite there being no initial difference in diagnostic performance, suggesting that providing students with conceptual frameworks to organize learning might aid knowledge retention and transfer [14]. Another important suggestion from this study is that short-term performance might be a poor predictor of long-term performance and that the relationship between these two may be conditional upon the way in which learning takes place.

The aim of this study was to identify determinants of long-term knowledge structure, as assessed by concept-sorting. The first objective was to examine the change in knowledge structure in medical students over a one year time period, during which time they received no further formal teaching in the clinical presentation under study. The second objective was to examine the association between the use of a conceptual framework (diagnostic scheme) during learning and long-term knowledge structure.

## Methods

### Study design and sample

This was a prospective observational cohort study. In a previous study, 81 medical students from the first year medical school class at the University of Calgary taking the Renal Course completed a concept-sorting task on the clinical presentation of metabolic alkalosis [7]. In this present study we rated the knowledge structure of the same students one year after the Renal Course (referred to as long-term knowledge structure) using the same concept-sorting task as our previous study.

### Concept sorting

Concept sorting is an indirect measure of knowledge structure in which subjects are asked to divide a list of diagnoses (typically around 20), that might cause the clinical presentation of interest, into different categories. Subjects name the categories and subdivided them as many times as desired. The diagnostic groupings are displayed as a hierarchal network, which is assumed to represent knowledge structure of diagnoses within this clinical presentation. The names given to the diagnostic categories are assumed to represent the concepts upon which knowledge is structured. The knowledge structure of subjects is compared to an *a priori *'expert framework' of interrelated key concepts for this clinical presentation. Rating of knowledge structure is part qualitative and part quantitative, considering both the presence or absence of concepts and their interrelationship.

In a previous study using concept sorting on experts in metabolic alkalosis four key concepts in knowledge structure emerged [7]. These concepts were: metabolic alkalosis may be due to a combination of *alkali ingestion and reduced excretion *(renal failure); causes of metabolic alkalosis without renal failure can be categorized according to the *effective arterial blood volume *(EABV); causes of reduced EABV can be categorized according to the *site of fluid loss*; and causes of expanded EABV can be categorized according to the *levels of renin and aldosterone*. Figure 1 shows the conceptual framework for metabolic alkalosis in which the categories are organized around these concepts. Note that most of these concepts are conditional upon another concept, e.g., identification of the site of fluid loss is conditional upon the EABV being reduced. This framework is based upon the results of CMA of experts (Nephrologists) in the presentation of metabolic alkalosis.

**Figure 2:**
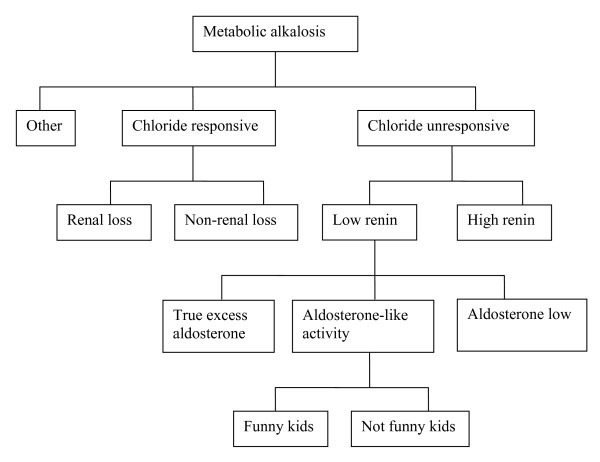
An example of a student with expert-type knowledge structure.

**Figure 3:**
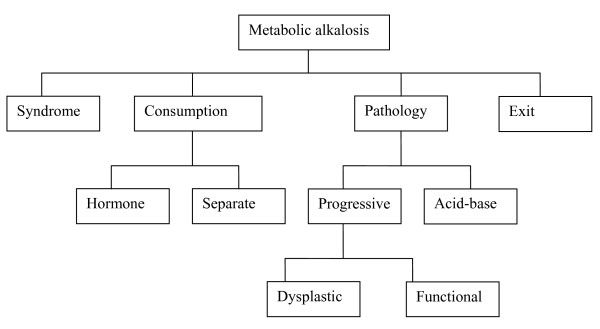
An example of a student with novice-type knowledge structure.

In the present study, as before, students' were considered to have expert-type knowledge structure if at least two of the four *a priori *concepts were identified in their knowledge structure, and these concepts had an appropriate interrelationship. Students failing to reach this threshold were considered to have novice-type knowledge structure. The threshold of two concepts was based on a previous study of concept sorting on 19 practicing nephrologists in which we found that each of these 'experts' used at least two of the *a priori *concepts [15]. Figure 2 shows the knowledge structure of a student considered to have expert-type knowledge structure, in which three of the four *a priori *concepts were identified (chloride-responsive is synonymous with reduced EABV). By contrast, Figure 3 shows a student considered to have novice-type structure. This knowledge structure contains several generic terms such as 'syndrome' and 'dysplastic', but also contains terms that could be interpreted as corresponding to the *a priori *concepts, such as 'consumption [but without reduced excretion]'. Note the use of the term 'hormone', which might refer to renin and aldosterone. Credit was not given for this concept as this is a conditional concept and is only appropriate in the setting where the EABV is expanded (here there is no mention of the EABV).

Two raters scored the concept-sorting independently and were blinded to the previous rating. During the time period between the two concept-sorting tasks the students did not receive any additional formal teaching in nephrology or the clinical presentation of metabolic alkalosis and were not evaluated on their knowledge of this presentation. We used the kappa statistic as a measure of inter-rater agreement for determining knowledge structure type by concept-sorting.

### Data analysis

To achieve the first study objective, a discordant pair analysis was performed (knowledge structure measured twice in the same individual one year apart cannot be considered as independent variables) using McNemar's test to identify the direction of change in knowledge structure, i.e., from novice-type to expert-type or vice versa. To achieve the second study objective, conditional logistic regression (measuring knowledge structure on more than one occasion requires an analysis of repeated measures) was performed where the dependent variable was long-term knowledge structure and the explanatory variables were short-term knowledge structure (i.e., immediately after instruction) and use of a diagnostic scheme by the student while learning. The possibility of an interaction between these variables was also considered. Statistical analyses were performed using STATA 8.0 software (Stata Corporation, College Station, Texas).

## Results

Thirty-four of the original 81 medical students completed the second concept-sorting task in the clinical presentation of metabolic alkalosis. Based on the questionnaire completed immediately after instruction, 24 of these students used a diagnostic scheme for metabolic alkalosis during learning and ten did not use a diagnostic scheme. The kappa statistic for inter-rater agreement for determining knowledge structure type (expert-type versus novice-type) by concept-sorting was 0.84 (p < 0.0001), representing 'almost perfect' agreement.

### Changes in the medical knowledge structure of students over time

Of the 34 students, 16 had the same knowledge structure type on repeat testing while 18 had a different knowledge structure type on repeat testing. There was a trend towards a greater number of students changing from expert-type to novice-type (12) than novice-type to expert-type (6), but the difference in discordant pairs did not reach statistical significance (p = 0.2).

### Variables associated with having expert-type knowledge structure one year later

By univariate analysis there was no association between short-term and long-term knowledge structure (odds ratio (OR) 0.88 [0.22, 3.46], p = 0.8). There was, however, increased odds of expert-type long-term knowledge structure in students who used a diagnostic scheme during learning (OR 12.6 [1.4, 116.0], p = 0.02).

By conditional logistic regression, a significant interaction was found between short-term knowledge structure and the use of a diagnostic scheme. We, therefore, performed stratified discordant pair analysis based upon the use of a diagnostic scheme. In the group who did not use a diagnostic scheme there was greater number of students changing from expert-type to novice-type than novice-type to expert-type (4 vs. 0, p = 0.046). There was no difference in the group that used the diagnostic scheme (8 vs. 6, p = 0.6). These data are shown in Tables 1 and 2, respectively.

## Discussion

In three previous studies, it was observed that knowledge structure, as evaluated by CMA, was a correlate of diagnostic performance [7-9]. It was also observed that using a diagnostic scheme during learning increased the odds of having expert-type short-term knowledge structure, as did the use of a diagnostic scheme by preceptors during the learning experience [7,9]. In the present study, the focus was on long-term knowledge structure and, in particular, the association between this dependent variable and two explanatory variables; short-term knowledge structure and the use of a diagnostic scheme during learning.

### Changes in the medical knowledge structure of students over time

Perhaps not surprisingly, the first finding in this study was that knowledge structure appears to change with time. In the group of students taken as a whole, there was a non-significant trend towards a greater number of students changing from expert-type to novice-type structure than vice versa. However, somewhat surprising was the fact that the change in knowledge structure was unpredictable, suggesting that variables other than decay over time may influence changes in knowledge structure.

### Variables associated with expert-type knowledge structure one year later

Two explanatory variables were considered as potential correlates of long-term knowledge structure; short-term knowledge structure and use of a diagnostic scheme during learning. On its own short-term knowledge structure was not observed to be a correlate of long-term knowledge structure, whereas use of a diagnostic scheme was. There was an interaction between these variables, suggesting that the association between short and long-term knowledge structure is conditional upon whether or not a diagnostic scheme was used during learning. In the group of students who did not use a diagnostic scheme there was a greater number of students who changed from expert-type to novice-type structure, whereas no such change was observed in the group using a diagnostic scheme. This observation suggests that students who developed expert-type knowledge structure during the period of instruction, and who use a diagnostic scheme in so doing, were more likely to keep expert-type knowledge structures than those who did not use a diagnostic scheme.

The apparent 'instability' of knowledge structure over time raises concerns regarding validity of studying knowledge structure. While these results suggest that short-term knowledge structure is not predictive of long-term knowledge structure, previous studies have shown that it is a correlate of [short-term] diagnostic performance. We would propose that evaluation of knowledge structure be used to try and explain the present [poor diagnostic performance] rather than predict the future [7-9]. In a recent study on diagnostic performance, Woods *et al *observed somewhat parallel results to these, i.e., that diagnostic performance immediately after instruction did not predict performance at a later time, whereas the method of learning was a correlate of later performance.

By virtue of the observational design of this study it was not possible to test the hypothesis that use of a diagnostic scheme during learning is responsible for the maintenance of expert-type knowledge structure. To test this hypothesis, a trial in which subjects are randomized to receive a diagnostic scheme or otherwise would be required. If such a benefit to the use of diagnostic schemes were confirmed, we would speculate that the mechanism by which diagnostic schemes might help maintain expert-type knowledge structure is by providing an organized and meaningful relationship between concepts. The way in which experts store knowledge of a clinical presentation [at least in the nephrology presentations that we have studied] is consistent with how knowledge is usually retrieved during the process of diagnostic reasoning (scheme-inductive reasoning) [16]. Thus, diagnostic schemes may provide internal encoding specificity that enhances the formation of, and retention of, expert-type knowledge structure [17].

### Cognitive strategies to facilitate learning and knowledge retention

In cognitive psychology the explicit use of cognitive strategies during educational encounters to facilitate learning and knowledge retention is well described and is referred to a 'reciprocal teaching' [10]. Conceptual frameworks, such as diagnostic schemes, are one of many strategies available. They have been studied widely in university students in a variety of domains and have also been studied to a lesser extent in medical students and health care professionals. While most studies show a short-term improvement in knowledge retention and problem solving, few studies have examined the effects of these strategies on longer-term knowledge retention. In the studies that evaluated knowledge after the initial learning period, the impact of conceptual frameworks appeared to be variable. This study adds to the existing body of literature by suggesting that the use of a conceptual framework to facilitate the development of expert-like knowledge structure may attenuate the loss of knowledge structure that was seen in students who did not use a conceptual framework.

### Study limitations

The present study has some important limitations. Results from an observational cohort study such as this are hypothesis generating and cannot be interpreted as showing that it is the use of a conceptual framework that facilitates (or 'causes') expert-type knowledge structure. As discussed above, to test this hypothesis a study in which subjects were randomized to receive the intervention (conceptual framework) or not, would be required. The small number of students who were studied at the two time points and the fact that only a single presentation was studied also limits generalizability of these results to other presentations. The stability of knowledge structure over time, including day-to-day, is not known and the results from this study would have been strengthened by several repeated measures of knowledge structure, rather than simply two measures separated by one year. In the present study we did not re-evaluate diagnostic performance in the clinical presentation of metabolic alkalosis and we cannot necessarily assume that the correlation between knowledge structure and diagnostic performance, evident in the short-term, persists. While the results of the clerkship examinations might have offered some overall assessment of diagnostic ability, given the importance of content specificity it was felt that this would not serve as an accurate assessment of diagnostic ability in the specific clinical presentation of metabolic alkalosis. Finally, the long follow-up period of one year, while advantageous in minimizing the influence of pre-examination cramming on knowledge structure, introduces the possibility of performance bias, although this would be likely to affect the groups equally.

## Conclusion

The results of this study suggest that the use of a diagnostic scheme by students may attenuate the loss of expert-type knowledge structure. We believe that this is a potentially exciting observation for medical educators that merits further investigation. This hypothesis should be tested in the setting of a randomized study comparing diagnostic schemes to alternative educational interventions.

## Competing interests

The author(s) declare that they have no competing interests.

## Authors' contributions

KN was involved in the data analysis and manuscript preparation. HM was involved in the study design. EW was involved in the study design and collected the data for the study. KM conceived of the study and was involved in data analysis and manuscript preparation. All authors read and approved the final manuscript.

## Pre-publication history

The pre-publication history for this paper can be accessed here:



## Figures and Tables

**Table 1:** Change in knowledge structure in students who did not use a diagnostic scheme.

		**Short-term knowledge structure**	
		
		**Novice-type**	**Expert-type**
**Long-term knowledge structure**	**Novice-type**	5	4
	**Expert-type**	0	1

**Table 2:** Change in knowledge structure in students who used a diagnostic scheme.

		**Short-term knowledge structure**	
		
		**Novice-type**	**Expert-type**
**Long-term knowledge structure**	**Novice-type**	2	8
	**Expert-type**	6	8
